# A Treatise on Electrolysis and Its Applications to Therapeutical and Surgical Treatment in Diseases

**Published:** 1886-11

**Authors:** 


A TREATISE ON ELECTROLYSIS AND ITS APPLICATIONS TO THERAPEUTICAL AND SURGICAL TREATMENT IN DISEASES. By Robert Amory, A. M., M. D. (Harvard), Fellow of Morse Medical Society; American Academy of Arts and Sciences; American Academy of Medicine; Boston Society of Medical Sciences, Corresponding Member of the Therapeutical Society of New York, etc.; formerly Professor of Physiology in the Medical School of Bowdoin College. New York: William Wood & Co., 56 and 58 Lafayette Place. 1886.

The constantly increasing scope for the use of electrolysis renders the appearance of this work most opportune, and those who wish to undertake this method of treatment have a long list of its applications laid before them. The first effect to follow the application of electricity is an infiltration with water of the subcutaneous tissue which overlies the hypertrophied growth, so that the flesh looks swollen. Secondarily a slow retrogism of the enlargement ensues with a shrivelled condition of the cutaneous covering. Repetition of the treatment is succeeded by a continued shrinking of the growth, which may be prolonged for several days after the enlargement has begun to retrograde. The principles involved, with the means of application, and the results in special cases, are well protrayed by the author, whose titles indicate his proficiency.

